# In vitro antioxidant and antiproliferative activities of plants of the ethnopharmacopeia from northwest of Mexico

**DOI:** 10.1186/1472-6882-13-12

**Published:** 2013-01-10

**Authors:** Manuel Jiménez-Estrada, Carlos Velázquez-Contreras, Adriana Garibay-Escobar, Davisela Sierras-Canchola, Ricardo Lapizco-Vázquez, Carolina Ortiz-Sandoval, Armando Burgos-Hernández, Ramón Enrique Robles-Zepeda

**Affiliations:** 1 Departamento de Productos Naturales, Instituto de Química, Universidad Nacional Autónoma de México, Cd. Universitaria, México, Distrito Federal, 04510, México; 2 Departamento de Ciencias Químico Biológicas, División de Ciencias Biológicas y de la Salud, Universidad de Sonora, Blvd. Luis Encinas y Rosales Hermosillo, Sonora, 83000, México; 3 Departamento de Investigación y Posgrado en Alimentos, División de Ciencias Biológicas y de la Salud, Universidad de Sonora, Encinas y Rosales Hermosillo, Sonora, 83000, México

**Keywords:** DPPH, Total phenol content, Flavonoids, Methanolic plants extracts, Medicinal plants Mexican

## Abstract

**Background:**

The aim of this study, is to investigate the in vitro antioxidant activity, the total phenols content, the flavonoids content and the antiproliferative activity of methanolic extracts of the plants: *Krameria erecta*, *Struthanthus palmeri*, *Phoradendron californicum*, *Senna covesii* and *Stegnosperma halimifolium*, used by different ethnic groups from northwestern Mexico in the treatment and cure of various diseases.

**Methods:**

The in vitro antioxidant activity was measured by 2,2-diphenyl-1-picrylhydrazyl (DPPH) and Ferric Reducing/Antioxidant Power assay (FRAP), the total phenols content was measured by Folin–Ciocalteau assay, the flavonoids content by the AlCl_3_ colorimetric method and the antiproliferative activity (line cells HeLa, RAW 264.7, M12A^k^.C3.F6 and L929) using MTT method.

**Results:**

The *K. erecta* extract showed the higher radical scavenging activity (67.88%), antioxidant activity by FRAP (1.41 mg Trolox Eq), the highest total phenols content (598.51 mg Galic Acid Eq/g extract), the highest flavonoids content (3.80 mg Quercetin Eq/g extract) and the greatest antiproliferative activity in a dose dependent manner against most Cell line evaluated. A positive correlation was found between the antioxidant activity and the flavonoids content.

**Conclusions:**

This study is the first report on the antioxidant and antiproliferative activities of the five species evaluated. The results demostrate that there is a positive correlation between antioxidant activity and the flavonoids content, indicating that these type of polyphenols could be the major contributors to the observed antioxidant activity in the evaluated plant extracts. Of the extracts evaluated, that of *Krameria erecta* showed the greatest antioxidant and antiproliferative activities, a discovery that makes this species a promising candidate for future research.

## Background

The knowledge of traditional medicine has always guided the search for new cures. In spite of the advent of modern high throughput drug discovery and screening techniques, traditional knowledge systems have given clues for the discovery of valuable drugs [1,2]. The medicinal use of natural products derived from plants, animals or microorganisms, has a long history.

Free radicals, produced as a result of normal biochemical reactions in the body, play important role in the human body and become harmful only when they are produced in high amounts. Free radicals are implicated in cancer, atherosclerosis, aging, immunosuppression, inflammation, ischemic heart disease, diabetes, hair loss, and neurodegenerative disorders such as Alzheimer’s disease and Parkinson’s disease [3-5]. The human body possesses innate defense mechanisms to counter free radicals in the form of enzymes such as superoxide dismutase, catalase, and glutathione peroxidase. Vitamin C, vitamin E, selenium, β-carotene, lycopene, lutein and other carotenoids have been used as supplementary antioxidants. Plant secondary metabolites such as flavonoids and terpenoids also play an important role in the defense against free radicals [6-8].

Free radicals, produced in oxidation processes, are essential for the production of energy to fuel biological processes in most living organisms. However, the excessive production of free radicals, such as superoxide radicals, hydroxyl radicals, hydrogen peroxide and nitric oxide has been associated with carcinogenesis, coronary heart disease, and many other health issues [9]. Cancer is a leading cause of death and may result from chronic injury to the epithelium by oxidants and other carcinogens.

From about 250,000 species of plants worldwide, all with potential to obtain natural products for the development of new drugs, only 1% of tropical species have been studied for their pharmaceutical potential [10]. About 4,000 species of Mexican plants have medicinal attributes, meaning roughly that one in seven species has a healing property. However it is estimated that the chemical, pharmacological and biomedical validations have been carried out in only 5% of these species [11].

In the state of Sonora in northwestern Mexico, it is estimated that the various indigenous groups used for medicinal purposes, about 450 different plants, including native and introduced species [12]. The aim of this work was to investigate the antiproliferative and antioxidant properties of five wild plants from northwest Mexico and used in the ethnopharmacopeia from state of Sonora, Mexico.

## Methods

### Plant samples

Wild plants were selected based upon their reported traditional use for the treatment of several diseases. General characteristics of the plant species used in this study are described in Table 1. The plants were collected in a region of the state of Sonora, northwest of México (Ranch “El Chaparral”, 111º 09’ 09” W; 28º 18’ 28” N), during February 2008. The plants were taxonomically identified by Professor Jesús Sánchez Escalante at the Universidad de Sonora Herbarium and a voucher of classification was assigned (Table 1).

**Table 1:** List of plants of the ethnopharmacopeia from Northwestern Mexico screened for the in vitro antiproliferative and antioxidant activities

**Plant**	**Common Name**	**Voucher**	**Family**	**Medicinal Use**
*Krameria erecta* Willd. ex Schult	Cosahui	USON2008-9	Krameriaceae	Diarrhea, colitis, enteritis, stomach and intestinal cancer
*Phoradendron californicum* Nutt	Toji de mesquite	USON2008-1	Viscaceae	Diarrhea
*Struthanthus palmeri* Kuijt	Toji	USON2008-7	Loranthaceae	Diarrhea
*Stegnosperma halimifolium* Bentham	Chapacolor	USON2008-3	Phytolaccaceae	Headache, bite snake, rabies
*Senna covessii* (A. Gray) Irwin & Barneby	Hoja Sen	USON2008-5	Fabaceae	Chickenpox, diseases of kidney and liver, measles

### Preparation of plant extracts

The plant extracts were obtained based on the procedures described by Ruiz-Bustos et al. [13]. Plant extracts of *Krameria erecta*, *Struthanthus palmeri*, *Phoradendron californicum*, *Senna covesii* and *Stegnosperma halimifolium* were prepared from air-dried samples (100 g) finely ground with a Wiley mill (200 mesh) and extracted with methanol (1:10 wt/vol) at room temperature. Extractions were carried out over a 10-day period with brief manual shaking twice daily. The extracts were passed through filter paper, dried under reduced pressure at 45°C, and weighed. These “crude” methanolic extracts were dissolved in dimethyl sulfoxide to a final concentration of 10 mg of dry matter/mL. All extracts were stored to −4°C in amber glass vials until use.

### Free-radical scavenging activity (DPPH assay)

Free-radical scavenging activity was measured as described previously with minor modifications [14]. Briefly, plant extract samples dissolved in absolute ethanol (0.6 mL) were mixed with an equal volume of a DPPH solution (300 μM) followed by vigorously vortexing for 10 s. After 30 min of incubation at room temperature in the dark, absorbance of the plant extract samples /DPPH solution was read at 517 nm in a spectrophotometer. The scavenging activity was determined by comparing the absorbance with that of the blank containing only DPPH and ethanol. Results were expressed as a percentage decrease with respect to control values. Plant extract samples and Trolox (used as antioxidant standard) were evaluated at different concentrations (12.5–100 μg/mL).

### Ferric reducing antioxidant power assay (FRAP assay)

The FRAP assay was carried out according to Benzie and Strain with some modifications [15]. The stock solutions included 300 mM acetate buffer (3.1 g C_2_H_3_NaO_2_·3H_2_O) and 16 mL acetic acid), pH 3.6, 10 mM TPTZ (2, 4, 6-tripyridyl-s-triazine) solution in 40 mM HCl, and 20 mM FeCl_3_·6H_2_O solution. The fresh working solution was prepared by mixing 25 mL acetate buffer, 2.5 mL TPTZ solution, and 2.5 mL FeCl_3_·6H_2_O solution. Plant extract samples (50 μL, 1 mg/mL) were mixed with 1.5 mL of the FRAP solution and allow to react for 5 min in the dark. Readings of the colored product [ferrous tripyridyltriazine complex] were then taken at 593 nm. The results were expressed as milligrams of trolox equivalents (mg TE/g).

### Determination of total phenols content

The total phenolic content of plant extracts was determined using the Folin-Ciocalteu reagent as described previously [16]. Briefly, 40 μL of plant extract samples (1 mg/mL) were diluted with distilled water (300 μL). Eighty microlitres of Folin-Ciocalteu reagent and 120 μL of a 20% sodium carbonate solution (w / v) were added. The volume was brought up to 1 mL with distilled water. The sample was left for 2 h and the absorbance was measured at 760 nm in a spectrophotometer. Based in a curve type realized previously, the results were expressed as milligrams of galic acid equivalents (mg GAE/g).

### Determination of total flavonoid content

The total flavonoids were determined using a colorimetric method as described previously [17]. Briefly, 0.5 mL of the plant extract (1 mg/mL) was diluted with 2 mL of distilled water. Then, 0.15 mL of a 5% NaNO_2_ solution was added to the mixture. After 6 min, 0.15 mL of a 10% AlCl_3_ solution was added and the mixture was allowed to stand for 6 min; 2 mL of 1M NaOH were added and the total mixture was made up to 5 mL using distilled water. The solution was mixed well and was allowed to stand for 15 min. Then, the absorbance was measured at 510 nm in a spectrophotometer. The results were expressed as milligrams of quercetin equivalents (mg QE/g).

### Cell lines and cell culture

Cell lines NCTC clone L929 (normal subcutaneous connective tissue) and HeLa (human cervix carcinoma) were purchased from the American Type Culture Collection (ATCC, Rockville, MD). The M12A^k^.C3.F6 (murine B-cell lymphoma) and RAW (macrophage, Abelson murine leukemia virus transformed) cells lines were kindly provided by Dr. Emil R. Unanue (Department of Pathology and Immunology, Washington University in St. Louis, MO). All cell cultures were carried out in Dulbecco’s modified Eagle’s medium (DMEM) supplemented with 5% heat inactivated fetal calf serum and grown at 37°C in an atmosphere of 5% CO_2_.

### Cell proliferation assay

To evaluate the effect of plant extracts on the proliferation of four cell lines, cell proliferation was determined using the standard MTT assay (3-(4,5-dimethylthiazol-2-yl)-2,5-diphenyltetrazolium bromide) [18]. Briefly, 10,000 cells (50 μL) were plated into each well of a flat 96 well plate. After 12 h incubation at 37°C in an atmosphere of 5% CO_2_ to allow cell attachment, the cell cultures were incubated with 50 μL of medium containing various concentrations of either crude extract or fraction, and the cell cultures were incubated for 48 h. The crude extract or fraction was first pre-suspended in DMSO and then diluted in DMEM media. Control cell cultures were incubated with DMSO (final concentrations of DMSO 0.06%-0.5%). Control cell cultures did not show any evidence of cell damage. In the last 4 h of the cell culture, 10 μL of MTT stock solution (5 mg/mL) were added to each well. Formazan crystals were dissolved with acidic isopropanol, and the plates were read in an ELISA plate reader, using a test wavelength of 570 nm and a reference wavelength of 630 nm. Plates were normally read within 15 min of adding isopropanol. The antiproliferative activity of extracts was reported as IC_50_ values (IC_50_ was defined as the concentration of extract evaluated and that to inhibit cell proliferation by 50%).

### Statistical analysis

All data were expressed as mean ± SD. Statistical analysis was performed by ANOVA procedures. A post-hoc test (Tukey) was carried out when the differences shown by data were significant (*p* <0.05). SPSS (version 16.0) statistical program was used for all analysis included the Pearson’s correlation coefficient.

## Results and discussion

There are no previous reports on the antioxidant activity, total phenols content, flavonoid content and antiproliferative activity of the five species reported here.

### Antioxidant activity

The DPPH method use organic radicals [19] and the FRAP assay can react with iron (II) and SH-group containing antioxidants [15], so it is expected that using these two methods accurately reflects all of the antioxidants in a sample.

### DPPH radical scavenging activity

Antioxidants are compounds that can delay or inhibit the oxidation of lipids and other molecules by inhibiting the initiation or propagation of oxidizing chain reactions [20]. The antioxidant activity is believed to be mainly due to their redox properties, which play an important role in adsorbing and neutralizing free radicals, quenching singlet and triplet oxygen, or decomposing peroxides [21]. The DPPH radical scavenging assay, based in the use DPPH, a stable free radical that decreases significantly on exposure to proton radical scavengers has been used to evaluate the free radical scavenging activity in foods, plant medicinal extracts and natural antioxidants [22-24]. The free radical scavenging activity of *Krameria erecta*, *Struthanthus palmeri*, *Phoradendron californicum*, *Senna covesii* and *Stegnosperma halimifolium* methanolic extracts was assessed by DPPH assay. Trolox was used as control.

**Figure 1:**
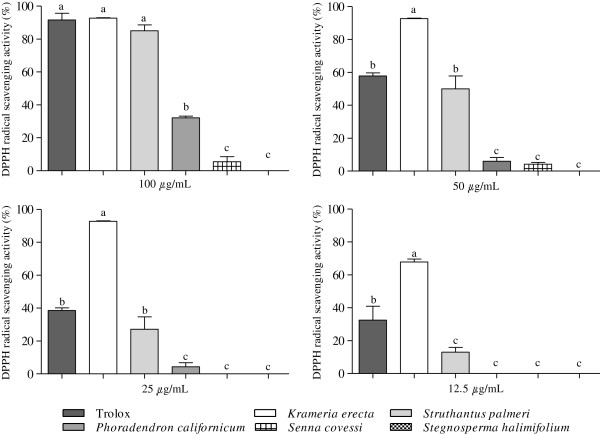
**Radical scavenging activity in the DPPH assay of *Krameria erecta*, *Struthanthus palmeri*, *Phoradendron californicum*, *Senna covesii *and *Stegnosperma halimifolium *methanolic extracts.** Trolox was used as control. The bars represent the standard deviation (mean ± SD; n = 3). Significant differences (p < 0.05) are indicated by different letters (a–c).

The results show that there is a dose-dependent radical scavenging activity (Figure 1). At all concentrations tested, the highest DPPH radical scavenging activity (%) was shown by *Krameria erecta* (92.74 ± 0.31) and *Struthantus palmeri* (85.13 ± 3.50) at 100 μg/mL, which were at the same level with the standard trolox. By contrast *Senna covessi* and *Stegnosperma halimifolium* showed no activity at any of the concentrations evaluated. At the lowest concentration tested (12.5 μg/mL), *K. erecta* was the one that showed higher radical scavenging activity (67.88%), with a significant difference (Tukey, p <0.05) with the rest of the extracts, including the control Trolox. The DPPH scavenging activity is arranged in the following descending order: *K. erecta* >*S. palmeri* >*P. californicum* >*S. covesii* >*S. halimifolium*.

The *Krameria erecta* specie is one of 17 belonging to the only genus of the Family Krameriaceae. It has been reported for this genus the presence of phenolic compounds like tannins and lignans and various biological activities such as hepatoprotective effect, antioxidant and anti-inflammatory [25-29]. The prevention of the chain initiation step by scavenging various reactive species such as free radicals is considered to be an important antioxidant mode of action [30]; in this context, *Krameria erecta* is likely to be acting through this mechanism.

### Ferric reducing antioxidant power assay (FRAP assay)

A FRAP method is based on the ability of an antioxidant to reduce (electron transfer) Fe^3+^ to Fe^2+^ ions in the presence of TPTZ (2,4,6-tris(2-pyridyl)-s-triazine), forming an intense blue Fe^2+^-TPTZ complex with an absorption maximum at 593 nm. The reaction is pH-dependent (optimum pH 3.6). The absorbance decrease is proportional to the antioxidant (reductant) content. Compounds that are active in Fe^3+^ reduction also stimulate the formation of OH^·^ (prooxidation activity) [31].

**Figure 2:**
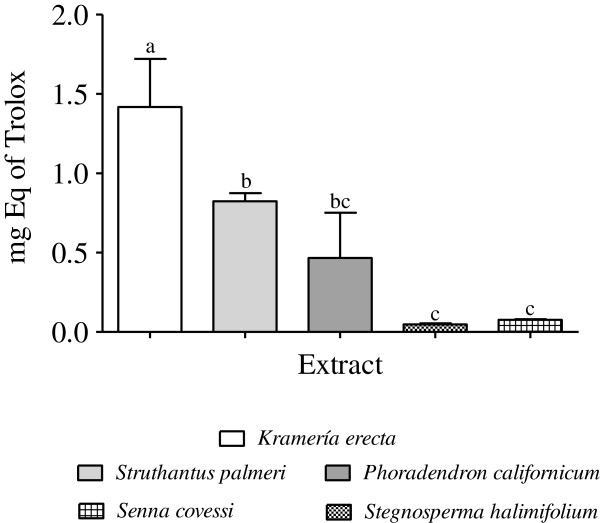
**Ferric reducing antioxidant power assay (FRAP assay) of *Krameria erecta*, *Struthanthus palmeri*, *Phoradendron californicum*, *Senna covesii *and *Stegnosperma halimifolium *methanolic extracts.** The results are expressed as mg Eq of Trolox. The bars represent the standard deviation (mean ± SD; n = 3). Significant differences (p < 0.05) are indicated by different letters (a–c).

**Figure 3:**
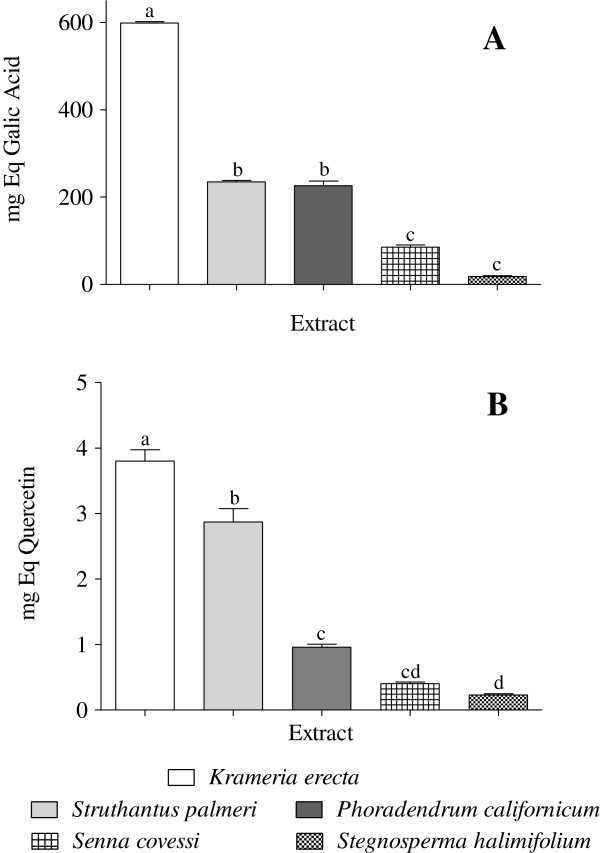
**A) Total phenols content (expressed as mg Eq of galic acid), B) total flavonoids content (expressed as mg Eq of quercetin) of *Krameria erecta*, *Struthanthus palmeri*, *Phoradendron californicum*, *Senna covesii *and *Stegnosperma halimifolium *methanolic extracts.** The results are. The bars represent the standard deviation (mean ± SD; n = 3). Significant differences (p < 0.05) are indicated by different letters (a–d).

The FRAP assay results of the five methanolic extracts expressed as mg equivalent of trolox are shown in the Figure 2. In accordance with findings from the DPPH assay, all extracts showed a similar antioxidant activity by FRAP assay, the *Krameria erecta* extract showed the highest reducing power among the extracts with 1.41 mg Trolox Eq (mg TE), followed by *Struthantus palmeri* (0.82 mg TE). The FRAP values varied from 1.41 to 0.04 (mean 0.56) mg TE, with a significant difference (Tukey, p <0.05) with the rest of the extracts. The methanol extracts with lower antioxidant activity by FRAP, were from *Stegnosperma halimifolium* and *Senna covesii* with 0.047 and 0.075 mg TE, respectively. The extracts with lower antioxidant activity by both methods (DPPH and FRAP) were those with a lower totals phenols content and flavonoids (*S. halimifolium* and *S. covesii*, Figures 3A and B), which indicates that there is a relationship between the antioxidant activity and content of phenolic type compounds in both plant extracts.

Although the DPPH and FRAP methods have different molecular targets, we observe a high correlation between the results of both methods, with a correlation coefficient *r* = 0.9501 (Table 2).

**Table 2:** Pearson’s correlation coefficients (r) between the analysis parameters

	***Flavonoides***	***Total phenols***	***DPPH***	***FRAP***
Flavonoids	1			
Total phenols	0.895862508	1		
DPPH	0.983311003	0.844512931	1	
FRAP	0.976039557	0.965356286	0.950100916	1

### Total phenols content

Phenolic compounds are highly effective free radical scavengers and exhibit strong antioxidant activity. The contents of phenolic compounds determined spectrometrically by the Folin-Ciocalteu method of the different analyzed methanolic extracts, are presented in Figure 3A and values are expressed as mg Gallic Acid Eq/g extract (mg GAE/g extract). The total phenols content varied from 598.51 to 18.06 mg GAE/g extract. *Krameria erecta* extract had the highest total phenols content (598.51 mg GAE/g extract) with a significant difference (Tukey, p <0.05) with the rest of the extracts, followed by *S. palmeri* extract (234.69 mg GAE/g extract). The extract of *S. covesii* and *S. halimifolium* showed the lowest content of phenols with 85.11 mg GAE/g extract and 18.06 mg GAE/g extract, respectively. Similar results to those of *K. erecta* have been reported for other species such as *Polygonum aviculare* (677.4 GAE/g extract) [32], but it has a higher content than other species reported [33,34].

It is well known that plant polyphenols are widely distributed in the vegetal kingdom and that they are sometimes present in surprisingly high concentrations; such is the case of *K. erecta*. This plant, commonly known by the name of cosahui, is widely used by ethnic groups in northwestern Mexico, for the treatment of various gastrointestinal diseases such as diarrhea and stomach and intestinal cancer [35]. Antioxidants derived from plant extracts have been shown to have multiple biological activities including vasodilatatory, anti-inflammatory, antiviral, antiproliferative, and antimicrobial effects [16,36,37].

Since the structural features of phenolic compounds are commonly reported as responsible of the antioxidant activity, the measurements of phenols in extracts may be related to their antioxidant properties [38]. In this study, DPPH scavenging activity and FRAP activity of extracts showed similar trend with the result of total phenolic content, r=0.8445 and r=0.9653, respectively, indicating that these activities of extracts are highly related to the amount of phenolic compounds, especially flavonoids that present in the extracts (Table 2).

### Total flavonoid content

Flavonoids are the most common and widely distributed group of plant phenolic compounds, which are usually attributed to their antioxidant properties [34]. In this study, the total flavonoid content from different methanolic extracts was evaluated by AlCl_3_ colorimetric assay. Quercetin was used as a standard and the total flavonoid content was expressed in mg Quercetin Eq per gram of extract (mg QE/g extract). The results are showed in the Figure 3B.

Similar to that observed in total phenols content, *Krameria erecta* showed the highest content of flavonoids (3.80 mg QE/g extract), followed by *S. palmeri* (2.87 mg QE/g extract), with a significant difference (Tukey, p< 0.05). The lowest total flavonoid content was observed in *S. halimifolium* (0.23 mg QE/g extract). The total flavonoid content is arranged in the following descending order: *K. erecta* >*S. palmeri* >*P. californicum* >*S. covesii* >*S. halimifolium*.

Flavonoids are described as hydrogen donating antioxidants by virtue of the reducing properties of the multiple hydroxyl groups attached to their aromatic ring system, along with their ability to delocalize the resulting phenoxyl radical within the structure. It is recognized that polyphenolic flavonoids are able to scavenge different reactive oxygen radicals such as the hydroxyl and superoxide radicals [39]. In this study, it is likely that the antioxidant activity observed is related to the flavonoid content, since the results from both methods used to determine the antioxidant activity, DPPH and FRAP, the total flavonoid content showed a better correlation with r values of 0.9833 and 0.9760, respectively (Table 2). The correlation between flavonoids and total phenols was r=0.8958 (Pearson’s correlation coefficients, Table 2), due to this, flavonoids probably not the main component in total content of phenols.

### Cell proliferation assay

**Figure 4:**
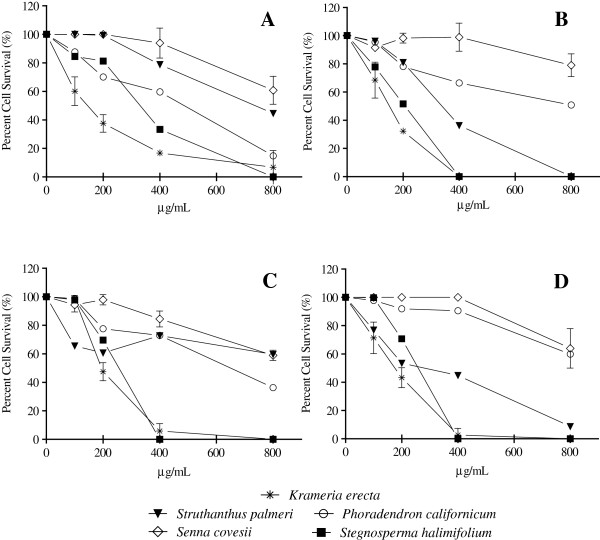
**Antiproliferative activity of the extracts on A) HeLa, B) RAW 264.7, C) M12A**^**k**^**.C3.F6 and D) L929 cells of *Krameria erecta*, *Struthanthus palmeri*, *Phoradendron californicum*, *Senna covesii *and *Stegnosperma halimifolium *methanolic extracts.** The bars represent the standard deviation (mean ± SD; n = 3).

The antiproliferative activity of the extracts on HeLa, RAW 264.7, M12A^k^.C3.F6 and L929 cells was evaluated using the MTT assay, which demonstrates mitochondrial activity of cells and is conventionally used as a measure of cell viability. The results are shown in Figure 4.

The extract of *K. erecta* showed the greatest antiproliferative activity in a dose dependent manner, being more active against HeLa and RAW 264.7 cells (IC_50_ <200 μg/mL), while at 400 μg/mL showed a 100% inhibition of cell growth. Additionally, to determine whether the extracts had unspecific cytotoxicity on any cell type, we also tested the effect on the growth of L929, a normal line of subcutaneous connective tissue. On this cell line, *K. erecta* extract showed an IC_50_ of <200 μg/mL, and at 400 μg/mL completely inhibited growth (Figure 4).

The extract of *S. halimifolium* showed an IC_50_ for antiproliferative activity of between 200–400 μg/mL with a 100% inhibition of cell growth at 400 μg/mL for RAW 264.7, M12Ak.C3.F6 and L929 cell lines. The extract of *S. palmeri*, presented an IC_50_ of between 200–400 μg/mL only for cell lines RAW 264.7 and L929. The remaining two extracts, *P. californicum* and *S. covesii* showed no significant antiproliferative activity at concentrations evaluated in this study.

Lignans are involved in the inhibition of phospholipase Cγ1, a key enzyme involved in signaling pathways and essential for proliferation by mitogens [40]. Lignan-type compounds have been isolated from different species of *Krameria*[27,28,41,42]. Considering the antioxidant activity, the content of total phenols, flavonoids and antiproliferative activity observed in the methanolic extract of *K. erecta*, it is likely that these biological activities are related to the presence of lignan-type compounds already observed in other species of the genus.

In addition to the significant antiproliferative activity, as mentioned above, the methanolic extract of *K. erecta* presented a great antioxidant activity. The events of oxidative cell damage are often correlated with the oxidative stress, so that the presence of both properties in this extract could be beneficial for preventive or therapeutic purposes. Since the preliminary results reported here are very promising, it is very important to explore the isolation of pure compounds responsible for these activities.

## Conclusions

This study is the first report on the antioxidant and antiproliferative activities of the five species evaluated. The results demonstrates that there is a positive correlation between antioxidant activity and the flavonoids content, indicating that these type of polyphenols could be the major contributors to the observed antioxidative activity in the evaluated plant extracts. Of the extracts evaluated, that of *Krameria erecta* showed the greatest antioxidant and antiproliferative activities, a discovery that makes this species a promising candidate for future research.

## Competing interests

The authors declare that they have no competing interests.

## Authors’ contributions

MJE, CVC conceived the study, analysis of data and drafting of the manuscript. AGE, DSC, RLV, COS, were involved in cell culturing, MTT, DPPH and total phenolic content assays. ABH was involved in the flavonoids assay and analysis of data. RERZ, was responsible for conception and design, acquisition of data, analysis and interpretation of data and drafted the manuscript. All authors have read and approved the final manuscript.

## Pre-publication history

The pre-publication history for this paper can be accessed here:

http://www.biomedcentral.com/1472-6882/13/12/prepub
